# Structural and Functional Characterization of *Camelus dromedarius* Glutathione Transferase M1-1

**DOI:** 10.3390/life12010106

**Published:** 2022-01-12

**Authors:** Fereniki Perperopoulou, Nirmal Poudel, Anastassios C. Papageorgiou, Farid S. Ataya, Nikolaos E. Labrou

**Affiliations:** 1 Laboratory of Enzyme Technology, Department of Biotechnology, School of Applied Biology and Biotechnology, Agricultural University of Athens, 75 Iera Odos Street, GR-11855 Athens, Greece; perperopoulouf@aua.gr; 2 Turku Bioscience Centre, University of Turku and Åbo Akademi University, 20521 Turku, Finland; nirpou@utu.fi (N.P.); anapap@utu.fi (A.C.P.); 3 Biochemistry Department, College of Science, King Saud University, P.O. Box 2455, Riyadh 11451, Saudi Arabia; fataya@KSU.EDU.SA

**Keywords:** abiotic stress, biotic stress, glutathione transferase, herbicide detoxification, pesticide determination, xenobiotics

## Abstract

Glutathione transferases (GSTs; EC. 2.5.1.18) are a large family of multifunctional enzymes that play crucial roles in the metabolism and inactivation of a broad range of xenobiotic compounds. In the present work, we report the kinetic and structural characterization of the isoenzyme GSTM1-1 from *Camelus dromedarius* (*Cd*GSTM1-1). The *Cd*GSΤM1-1 was expressed in *E. coli* BL21 (DE3) and was purified by affinity chromatography. Kinetics analysis showed that the enzyme displays a relative narrow substrate specificity and restricted ability to bind xenobiotic compounds. The crystal structures of *Cd*GSΤM1-1 were determined by X-ray crystallography in complex with the substrate (GSH) or the reaction product (S-p-nitrobenzyl-GSH), providing snapshots of the induced-fit catalytic mechanism. The thermodynamic stability of *Cd*GSTM1-1 was investigated using differential scanning fluorimetry (DSF) in the absence and in presence of GSH and S-p-nitrobenzyl-GSH and revealed that the enzyme’s structure is significantly stabilized by its ligands. The results of the present study advance the understanding of camelid GST detoxification mechanisms and their contribution to abiotic stress adaptation in harsh desert conditions.

## 1. Introduction

Mammals are exposed to many factors that can affect their functions or even cause their demise. Such factors can be xenobiotics, such as pesticides, environmental pollutants, and reactive oxygen species (ROS) [1,2]. These factors activate a detoxification mechanism, consisting of two phases (phase I and phase II) [3]. In phase II reactions, glutathione transferases (GSTs) are involved and encoded by multiple genes [4]. Upon entering the living cell, xenobiotic and toxic compounds may undergo GST-mediated conjugation with glutathione (GSH) in order to become more soluble and to be excreted by the cell [5].

GSTs, depending on their protein sequence and structure, are known as different superfamilies: cytosolic (cGSTs), mitochondrial (mGSTs), and microsomal (MGSTs) [6]. In mammals, cytosolic GSTs are further categorized, based on sequence similarity, into seven classes: alpha (a), zeta (Z), theta (T), mu (M), pi (P), sigma (S), and omega (O) [7]. Similarity of GSTs within a class is greater than 60 %, whereas interclass sequence similarities go up to 30 % [8]. However, all cytosolic GSTs share a universal GST-fold [9]. They are dimeric enzymes, usually from identical chains, but heterodimers made of two different chains are also found [10]. Two domains are recognized in each monomer: the conserved N-terminal thioredoxin-like domain, containing the GSH binding site (G-site), and the less conserved C-terminal domain, containing the xenobiotic binding site (H-site) [11].

The one-humped camel, *Camelus dromedarius*, plays an important socio-economic role in many countries in Asia, Africa, and the Arabian Peninsula [12]. In fact, this animal is of great economic importance as it is used for milk, meat, and fleece production, while many countries use camels for transport, tourism, and races [13]. The camelid genes involved in biotransformation processes and in mechanisms of adaptation in the arid lands are of great importance in the research domain [1,14]. The structural and stability properties of *Cd*GSTM1-1 were recently investigated [14]. The enzyme exists in a dimeric state at pH 7.0, however, at acidic pH 2.0, it appears as a monomeric protein. The dimeric form exhibits relatively less thermal stability compared to the monomeric form. Although the majority of proteins are less stable below pH 5.0 and above pH 10.0, the relatively higher stability of the monomer of *Cd*GSTM1-1, compared to the dimer, may be the consequence of the large number of intra-chain interactions within the monomer [14].

The isoenzyme GSTM1-1 is mostly studied in humans for its association with many types of cancer [15]. Indeed, an overexpression of GSTM1-1 may limit the efficacy of many anticancer drugs [16]. Other studies support the association of GSTM1-1 with Parkinson’s disease, as well as other illnesses related to oxidative stress [17,18].

In this work, we studied the isoenzyme GSTM1-1 from *Camelus dromedarius* in order to understand its functional and catalytic role in the detoxification mechanism of *Camelus dromedarius*.

## 2. Materials and Methods

### 2.1. Materials

Reduced GSH, 1-chloro-2,4-dinitrobenzene (CDNB), ampicillin, and all of the other enzyme substrates were obtained from Sigma-Aldrich, (Saint Louis, MO, USA). Isopropyl 1-thio-β-galactopyranoside (IPTG) was obtained from AppliChem (Darmstadt, Germany). Yeast extract and peptone were obtained from Scharlau (Sentmenat, Barcelona Spain). All other chemicals were of analytical grade and were purchased from Merck (Darmstadt, Germany).

### 2.2. Methods

#### 2.2.1. Expression and Purification

The expression of recombinant *Cd*GSTM1-1 was performed according to the general protocol described in published procedures [19,20,21,22]. *E. coli* BL21 (DE3) cells harboring recombinant plasmid (pET-3aCdGSTM1-1) were grown at 37 °C in 1 L LB medium containing ampicillin (100 μg/mL). The expression of *Cd*GSTM1-1 was induced by isopropyl-1-thio-β-galactopyranoside (1 mM) when the absorbance at 600 nm was 0.6 and was further incubated for 4 h. Then, the culture was centrifuged (8000 × g for 10 min at 4 °C). The collected cells were resuspended in a potassium phosphate buffer (20 mM, pH 7), sonicated, and centrifuged for 5 min at 13,000 × g. The supernatant was used for the purification of *Cd*GSTM1-1 using affinity chromatography on GSH-Sepharose adsorbent [10]. SDS-PAGE (12% *w*/*w*) was used for evaluation of the protein purity.

#### 2.2.2. Assay of the Enzyme Activity, Protein and Kinetic Analysis

Enzyme assays were carried out in 1 mL of 100 mM potassium phosphate buffer with a pH of 6.5, according to Axarli et al. [10] and Perperopoulou et al., [1]. Determination of the *Cd*GSTM1-1 activity was performed by monitoring the formation of the conjugate between CDNB and GSH at 340 nm (ε = 9600 L × mol^−1^ × cm^−1^) for 120 s. One unit of enzyme activity is defined as the amount of enzyme that produces 1.0 μmol of product per minute under the assay conditions. Initial velocities were determined in triplicate and were corrected for the spontaneous reaction rates, when necessary. The Michaelis–Menten equation was fitted to the experimental data by nonlinear regression analysis using the GraphPad Prism version 7 (GraphPad Prism Software, Inc.) computer program. The protein concentration was determined by the Bradford assay [23].

### 2.3. Inhibition Analysis

The inhibition potency of different pesticides towards *Cd*GSTM1-1 was analyzed using 0.1 M potassium phosphate buffer, pH 6.5, according to Chronopoulou et al. [24]. The IC_50_ value of zoxamide was measured using the CDNB/GSH system in the presence of different concentrations of fungicide (0.01–10 μM). The IC_50_ value was determined using the GraphPad Prism version 7 (GraphPad Prism Software, Inc.) computer program.

### 2.4. Kinetic Inhibition Studies

Initial velocities for the *Cd*GSTM1-1 catalyzed reaction with GSH as the variable substrate (0.12–3.6 mM) were performed in a total volume of 1 mL mixture containing 0.1 M potassium phosphate buffer (pH 6.5), in the absence or in the presence of different concentrations of zoxamide (0.75 μΜ, 1.5 μΜ and 2 μΜ). With CDNB as the variable substrate (0.15–1.75 mM), the reaction mixture (total volume 1 mL) contained 0.1 M potassium phosphate buffer (pH 6.5) in the absence or in the presence of different concentrations of zoxamide (0.75 μΜ, 1.5 μΜ, and 2.5 μΜ). The apparent kinetic parameters were determined using the computer program the GraphPad Prism version 7 (GraphPad Prism Software, Inc.).

### 2.5. Thermal Stability

The thermal stability of *Cd*GSTM1-1 was investigated by incubating the purified enzyme at different temperatures (4 to 53.5 °C) in 0.1 M potassium phosphate buffer at a pH of 6.5. The samples of enzyme were incubated for 5 min and were subsequently assayed for their residual activity. T_m_ values were determined from the plot of the relative inactivation (%) versus temperature (°C). The T_m_ value corresponds to the temperature at which 50% of the initial enzyme activity is lost after heat treatment.

The thermal stability of *Cd*GSTM1-1 was also investigated using differential scanning fluorimetry (DSF) on an Applied Biosystems^®^ real-time PCR StepOne™ instrument, as described by Pouliou et al. [19]. Fluorescence monitoring was carried out at 4–99 °C at a rate of 1 °C/min. Melting temperatures (T_m_) were estimated using the Protein Thermal Shift™ Analysis Software (Applied Biosystems). Ligand-binding analysis was also achieved with DSF in the presence of different concentrations of GSH and S-hexyl-GSH (0.1 and 0.5 mM).

### 2.6. Crystallization

The protein solution was concentrated and buffer-exchanged using 10 mM HEPES, pH 7.0 and 150 mM NaCl. *Cd*GSTM1-1 was used at 10 mg/mL for crystallizations with the hanging drop vapor diffusion method. Drops consisted of 2.0 μL of protein solution mixed with 2 μL of reservoir (100 mM Tris-HCl, 24% (*w/v*) PEG 4000, 0.2 M sodium formate, pH 8.6). The plates were left for equilibration at 16 °C. S-p-nitrobenzyl-GSH, dissolved in 0.1 M KNa phosphate buffer, pH 7.0, was used at a final concentration of 2.5 mM. Crystallizations without the addition of S-p-nitrobenzyl-GSH were carried out in parallel under the same conditions. Data were collected on the P13 beamline at PETRA III (DESY, Hamburg) at a cryogenic temperature (100 K) from crystals flash frozen with liquid nitrogen in the presence of 20% (*v*/*v*) glycerol as cryoprotectant.

### 2.7. Structure Solution and Refinement

The structure of *Cd*GSTM1-1 was solved by molecular replacement using the ligand-free *h*GSTM1-1 (PDB id 1gtu) as the search model. Automated molecular replacement through MrBump [25] was employed, which produced a solution with an *R*_work_/*R*_free_ of 0.29/0.33 after 30 cycles of restrained refinement in REFMAC. Refinement was subsequently carried out with Phenix (v. 1.17.1-3660) [26], using simulated annealing at 1000 K and maximum likelihood as the refinement target.

## 3. Results and Discussion

### 3.1. Purification and Kinetic Analysis

**Figure 1:**
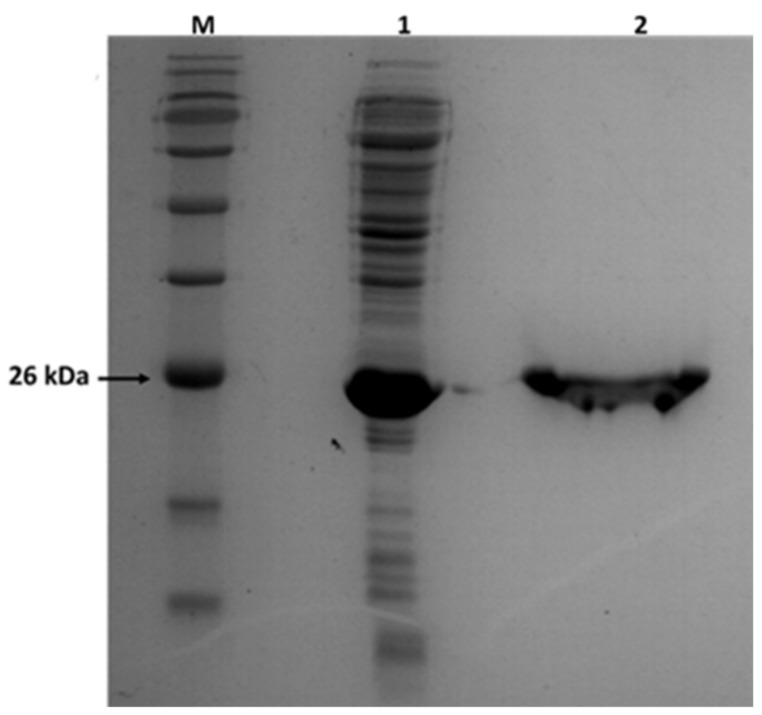
SDS-PAGE analysis of *Cd*GSTM1-1 purification. Protein bands were stained with Coomassie Brilliant Blue R-250. M: MW markers; Lane 1: *E. coli* BL21 (DE3) crude extract after induction with 1 mM IPTG; Lane 2: *Cd*GSTM1-1purified by affinity chromatography on GSH-Sepharose (elution with 10 mM GSH).

Recombinant *Cd*GSTM1-1 was purified in a single-step by affinity chromatography using a GSH-Sepharose column as the adsorbent (Figure 1). The substrate specificity of the purified enzyme was assessed using a panel of model GST substrates, and the results are listed in Table 1. The enzyme appears to accept few electrophile compounds as substrates, compared to other GSTs [1,5,27]. Noteworthy, *Cd*GSTM1-1 lacks hydroperoxidase activity when using tert-butyl hydroperoxide and cumene hydroperoxide as the substrates. This is in contrast to human enzyme GSTM1-1, which displays significant hydroperoxidase activity towards phosphatidylcholine hydroperoxide [28]. Similarly, the enzyme is inactive towards α,β-unsaturated ketones such as ethacrynic acid and trans-4-phenyl-3-buten-2-one. On the other hand, *Cd*GSTM1-1 exhibits a high activity towards the isothiocyanate analogues allylisothiocyanate and phenethylisocyanate. Similar catalytic efficiency towards isothiocyanate analogues has also been observed for the human GSTP1-1 and GSTM1-1 [29]. The enzyme catalyzes the nucleophilic reaction of the thiol group of GSH to the electrophilic isothiocyanate group, giving dithiocarbamates [30]. The enzyme efficiently accepts both phenethylisocyanate and allylisothiocyanate as a substrate. The high specific activity suggests that GSTM1-1 contributes significantly in the metabolic disposition of isothiocyanates in camels. *Cd*GSTM1-1 also displays high sulphanilamidase activity with the model substrate sulphanilamide, similar to the human enzyme [31].

Analysis of the thiol specificity was achieved using GSH analogues (Table 1). The results showed that alternative thiols (e.g., γ-Glu-Cys, Cys-Gly, cysteine, and N-acetyl-L-cysteine) cannot replace the natural substrate GSH in the CDNB/GSH conjugation reaction, suggesting a very specific mechanism in *Cd*GSTM1-1 catalysis or in GSH molecular recognition.

**Figure 2:**
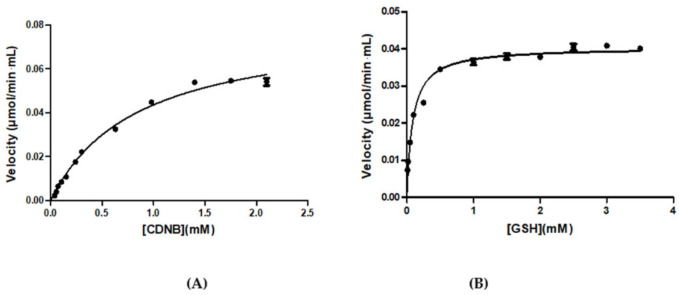
Kinetic analysis of *Cd*GSTM1-1 using the CDNB as a variable substrate (**A**) and GSH at a fixed concentration. Kinetic analysis of *Cd*GSTM1-1 using GSH as a variable substrate (**B**) and CDNB at a fixed concentration. The initial velocity values (y-axis) were plotted against the substrate concentration (x-axis), and a Michaelis-Menten nonlinear regression analysis was performed.

Steady-state kinetic analysis of the *Cd*GSTM1-1 using the model substrate system CDNB/GSH was achieved, and the results are illustrated in Figure 2. The kinetic parameters are listed in Table 2. The enzyme displays different kinetic parameters compared to the human GSTM1-1 enzyme [15]. *Cd*GSTM1-1 exhibited a 3.5-fold lower K_m_ for GSH and 4-fold higher K_m_ value for CDNB, suggesting a different catalytic function or biological role for the *Cd*GSTM1-1. This peculiar kinetic behavior of the *Cd*GSTM1-1, supported by its restricted xenobiotic substrate specificity, as well as the high K_m_ value for CDNB, prompted us to investigate its ability to bind other xenobiotic compounds.

### 3.2. The Ability of CdGSTM1-1 to Bind Xenobiotic Compounds

**Figure 3:**
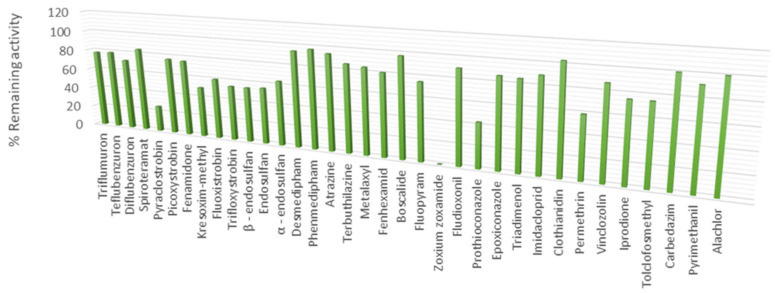
Inhibition of the *Cd*GSTM1-1 activity by pesticides. In the absence of pesticides, the enzyme activity was considered to be 100%. The results represent the means of the triplicate determinations, with less than 5% variation in all cases.

A well-known property of GSTs from all classes is their ability to bind hydrophobic compounds in a non-substrate manner, facilitating their transport or storage within the cell [32,33]. This feature of GSTs is termed ligandin function, and the ligand-binding site is either part of the substrate binding site (e.g., located within the H-site overlapping both the G- and H-site) or is positioned within the subunits interface. The ability of *Cd*GSTM1-1 to bind different xenobiotic compounds, such as pesticides, was assessed by measuring the inhibition of the enzyme’s activity by xenobiotics. From the results illustrated in Figure 3, it is evident that *Cd*GSTM1-1 displays a restricted ability to bind strongly xenobiotic compounds, in agreement with its relative narrow substrate specificity (Table 1). All but the fungicide zoxamide appear to be weak inhibitors towards *Cd*GSTM1-1, although the compounds were selected to possess significant differences in structure, size, or polarity. Most xenobiotics displayed a relative low inhibition potency (<50%) at 100 μΜ concentration, and therefore the fungicide zoxamide was selected for further study. The restricted ability of *Cd*GSTM1-1 to bind xenobiotics presumably reflects a biological role for *Cd*GSTM1-1 that is probably linked rather to detoxification and not to storage or the transfer of other molecules.

**Figure 4:**
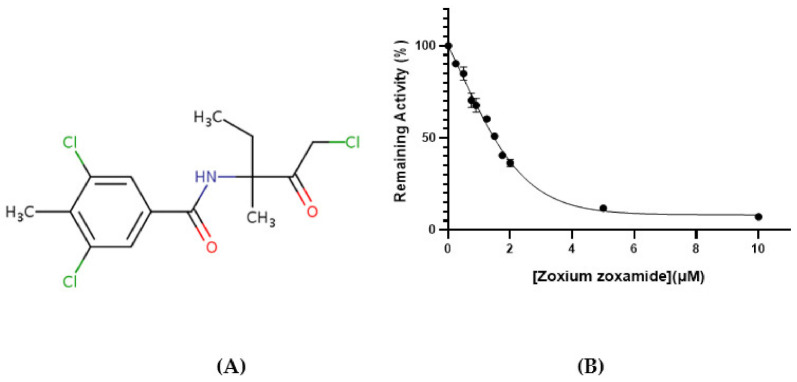
(**A**) The structure of zoxamide. (**B**) Concentration−response graph for the determination of the IC50 value for zoxamide with *Cd*GSTM1-1. The graph is produced using GraphPad Prism 7.

**Figure 5:**
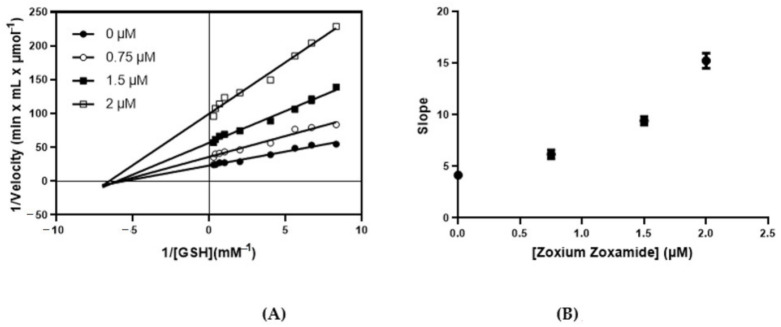
Kinetic inhibition analysis of *Cd*GSTM1-1 by zoxamide. Inhibition kinetics of *Cd*GSTM1-1 by zoxamide using GSH as a variable substrate. (**A**) The Lineweaver−Burk graphs of *Cd*GSTM1-1 inhibition at different concentrations of zoxamide (○ 0, ● 0.75, □ 1.5, and ■ 2 μM). (**B**) Secondary plot derived from the data of plot A. The graphs are created using the GraphPad Prism version 8 program (GraphPad Prism Software, Inc.).

**Scheme 1:**
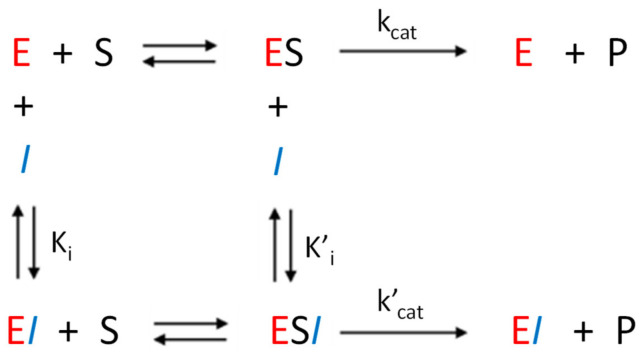
Partial mixed type inhibition model for the zoxamide against *Cd*GSTM1-1. When *Cd*GSTM1-1 (red, E) is inhibited by zoxamide (blue, *I*), there are two pathways: *I* can bind to either the free enzyme or the Michaelis−Menten complex (ES). Under these conditions, *Cd*GSTM1-1 is not fully inhibited and can produce product (P) either by ES or ESI, represented by rate constant k_cat_ or k_cat_′, respectively.

The concentration−response curve allowed forthe determination of IC_50_ = 1.49 ± 0.068 μΜ (Figure 4). Kinetic inhibition analysis was carried out in order to investigate the modality of inhibition by zoxamide and further to shine light on the location of its binding site on the enzyme’s structure. With GSH as a variable substrate, zoxamide displayed a mixed inhibition profile, as shown by the lines of the Lineweaver−Burk graph not intersecting the reciprocal velocity or GSH axes (Figure 5A). However, the slopes of each Lineweaver–Burk plot as a function of the inhibitor concentration follow a parabolic dependence, implying that the inhibition in this case is partially mixed (Figure 5B). The partial mixed-type inhibition model observed suggests that when the enzyme is inhibited by zoxamide, there are two pathways, as illustrated in Scheme 1. The inhibitor can bind to either the free enzyme or to the enzyme−substrates complex. Therefore, *Cd*GSTM1-1 is not fully inhibited and can produce a product either by ES or ESI. The inhibition constants K_i_ and K_i_’ were calculated from linear double reciprocal graphs, depicting 1/ΔSlope versus 1/[zoxamide] and 1/ΔIntercept versus 1/[zoxamide], constructed from the data of Figure 5B. The findings suggest that the inhibitor is able to bind to both the free enzyme and the enzyme−substrate complex, with inhibition constants of K_i_ = 0.074 ± 0.012 μΜ and K_i_’ = 0.018 ± 0.0026 μΜ, respectively.

**Figure 6:**
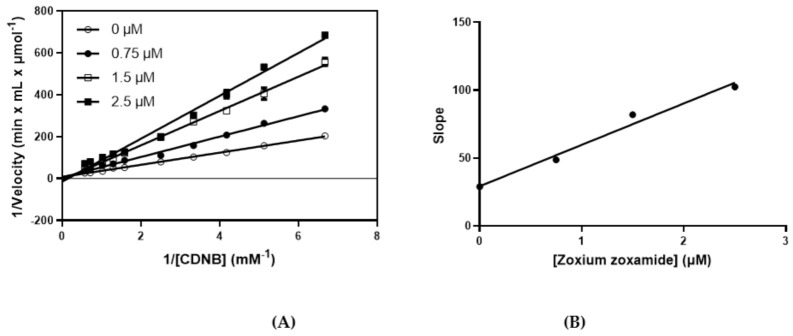
Inhibition kinetics of *Cd*GSTM1-1 with zoxamide using CDNB as a variable substrate. (**A**) The Lineweaver–Burk graphs of *Cd*GSTM1-inhibition at different concentrations of zoxamide (○ 0, ● 0.75, □ 1.5, and ■ 2.5 μM). (**B**) Secondary plot derived from data of plot A. The graphs were created using GraphPad Prism version 8 (GraphPad Prism Software, Inc.).

When using CDNB as a variable substrate, zoxamide showed purely competitive inhibition kinetics (K_i_ = 0.96 ± 0.29 μΜ) on the basis of linearity for both the double reciprocal Lineweaver–Burk graph (Figure 6A), at various concentrations of the fungicide, and its respective secondary graph (Figure 6B). This behavior suggests that this inhibitor competes with CDNB for the same binding site of the enzyme. The results of the kinetic analysis allowed for the elucidation of the ligandin binding site of the *Cd*GSTM1-1, and it was concluded that it is located into or overlaps with the H-site.

### 3.3. Structural Analysis of CdGSTM1-1 in Complex with GSH or S-hexyl-GSH

**Figure 7:**
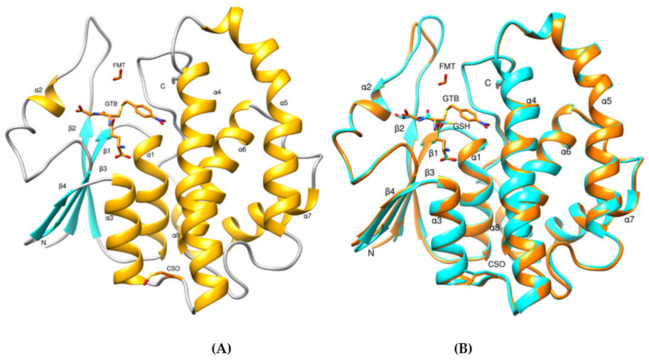
(**A**) Ribbon representation of molecule A of the *Cd*GSTM1 homodimer in complex with GTB. The molecule is colored according to secondary structure elements (β-strands in cyan and α-helices in orange). GTB, FMT, and CSO are shown as sticks and are colored according to atom type. (**Β**) Superimposition of GTB- (orange) and GSH-bound (cyan) *Cd*GSTM1 complexes.

The crystal structure of *Cd*GSTM1-1 in complex with GSH or S-(p-nitrobenzyl)-GSH was resolved by X-ray crystallography at 2.55 and 2.05 Å resolution, respectively (Table 3). In both complexes, there are four molecules of *Cd*GSTM1-1 per crystallographic asymmetric unit, resulting in two homodimers. The *Cd*GSTM1-1 homodimers are similar as in other mu class GSTs, with one active site per monomer. Each monomer consists of two domains: Domain I and Domain II. Domain I comprises residues 1−81 and domain II residues 89−217. Domain I is present at the N-terminal region of the molecule and is composed of a four-stranded β-pleated sheet flanked by three α helices. The N-terminal domains adopts a thioredoxin fold, which is highly conserved through GST classes. The G-site, responsible for binding GSH, is present in domain I. The G-site topology appears to be strictly conserved among all mu class GSTs. Domain II is found to be composed of five amphipathic α-helices and houses the H-site. The H-site is the more variable region of the protein and harvests the capacity of xenobiotics binding. Pro39 (*Cd*GSTM1-1 numbering) appears to be highly conserved in mu class GSTs and is responsible for providing a characteristic extended loop connecting β2 and α2 from domain I (Figure 7A). The ligands, S-(p-nitrobenzyl) glutathione (GTB), bound to the G-site (GSH part) and H-site (nitro-benzyl moiety) of *Cd*GSTM1-1, and formic acid (FMT), bound to the H-site, are shown in Figure 7A.

The superimposition of *Cd*GSTM1-1 structures with GSH-enzyme and GTB-enzyme complexes is presented in Figure 7B. The RMSD value between the co-ordinates of the backbone atoms of the monomers of the structures is 0.41 Å. The superimposed structures show that the overall configuration of the GSH moiety of GTB is identical to that of the GSH-enzyme complex (Figure 7B). Notably, a move of loop β2-α2 upon binding of S-p-nitrobenzyl-GSH (~4 Å) was observed, which may suggest an induced-fit mechanism to facilitate the binding of various substrates.

**Figure 8:**
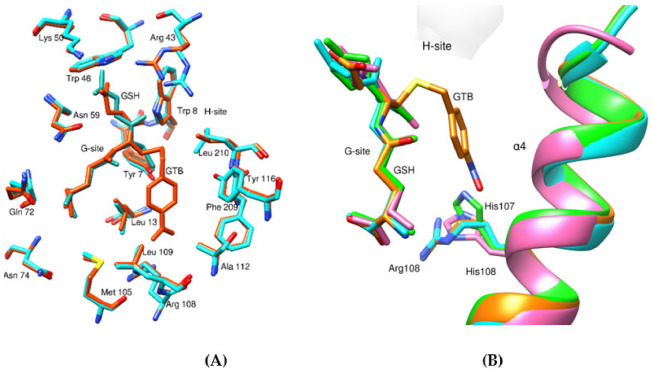
(**A**) Superposition of active-site residues taken from the GSH-bound (cyan) and GTB-bound (orange) *Cd*GSTM1 structure. (**B**) Substitution of *h*GSTM1 His107 (green) and *Lv*GSTM His108 residues (pink) with Arg108 in *Cd*GSTM1 (in orange and cyan for GTB- and GSH-bound complexes, respectively).

A conserved Tyr residue (Tyr7) found in the active site of the enzyme acts as the catalytic residue (Figure 8). Similarly, Trp8 and Leu13 are found to be conserved in all compared structures and are involved in the stabilization of GTB moiety in the active site via H-bonding interaction and pi-alkyl interaction with the nitro-benzyl ring, respectively. Furthermore, residues Tyr116, Gln72, Ser73, and Asp106 are highly conserved. The carbonyl oxygen of Gln72 and carboxyl group of Asp106 are likely to form an H-bond with the γ-Glu amino moiety of GTB. The amide group and side chain hydroxyl group of Ser73 are found to be H-bonded to the α-carboxyl group of GTB. The side chains of Arg43, Trp46, and Asn59 and the backbone carbonyl oxygen of Leu60 are found to interact with various polar groups of GTB and are conserved in the compared structures. Arg43 and Lys50 are located 3.09 Å and 3.52 Å, respectively, from the carboxylate glycine end of GTB, and are involved in the formation of a salt bridge with GTB contributing to the stability of GTB in the active site. Furthermore, His107 (hGSTM1-1 numbering), which has a pronounced influence on the catalysis of nucleophilic aromatic substitution reactions [34,35] in humans, has been substituted by Arg108 in all other compared rodent and avian mu class GSTs. It is also found that the guanidinium group of Arg108 is oriented away from the nitrobenzyl ring of GTB (Figure 8) and is only found to interact weakly via van der Waals forces with the substrate.

The H-site of the enzyme is in a proximity of the G-site. The H-site is composed of residues from the C-terminal region. The H-site, as in the other compared structures, is found to be mostly lined by hydrophobic residues. The hydrophobic residues contributing to the H-site of *Cd*GSTM1-1 are Met35, Arg43, Tyr116, Leu210, and Met212.

### 3.4. Structure Comparison with Human GSTM1-1 and Other GSTMs

**Figure 9:**
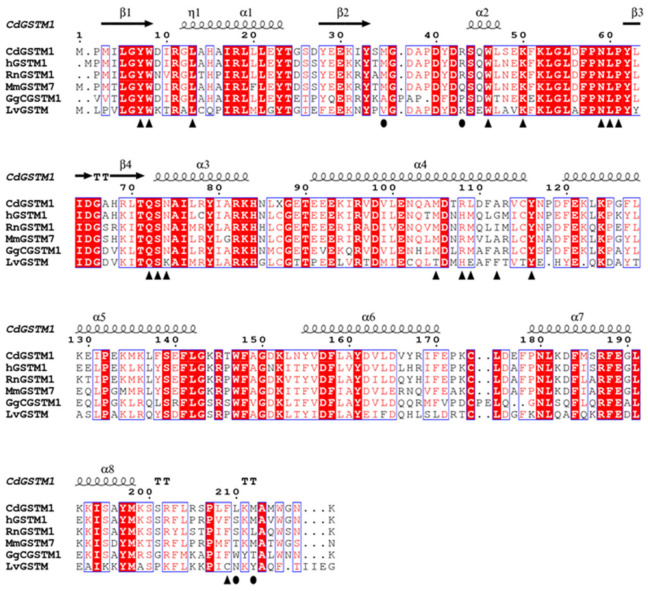
Structure-based sequence alignment of members of the mu family of GSTs. The secondary structure elements of *Cd*GSTM1 are displayed on top. The *Cd*GSTM1 residue numbering is shown above the alignment and the conserved areas are shaded. A column is framed if more than 70% of its physicochemical properties is similar. Residues involved in GTB binding are shown with triangles and the residues involved in FMT binding are shown in circles. *Homo sapiens h*GSTM1, PDB id 1xw6; *Rattus norvegicus Rn*GSTM1, PDB id 4gst; *Mus musculus Mm*GSTM7, PDB id 2dc5; *Gallus gallus Gg*CGSTM1, PDB id 1gsu; *Litopenaeus vannamei Lv*GSTM, PDB id 5an1.

**Figure 10:**
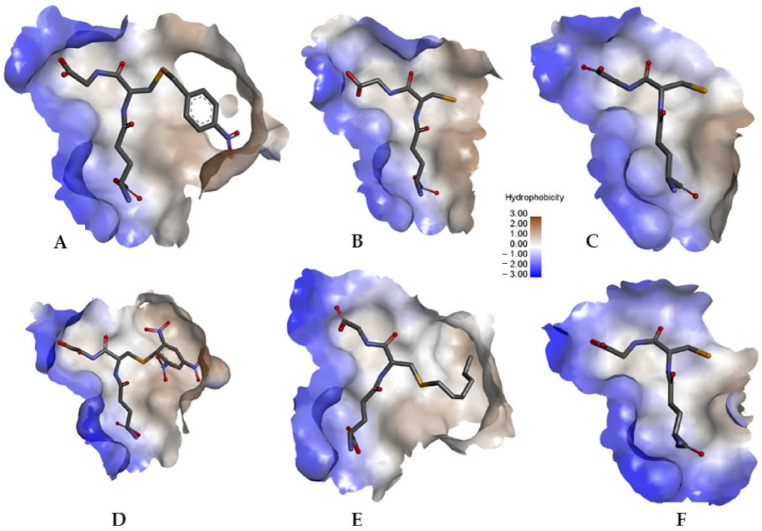
The active site of different mu class GSTs in the presence of various ligands. (**A**) GTB bound to *Cd*GSTM1. (**B**) GSH bound to *Cd*GSTM1. (**C**) GSH bound to *h*GSTM1 (PDB id 1xw6) [35]. (**D**) GTD bound to *Rn*GSTM1 (PDB id 4gst) [36]. (**E**) GTX bound to *Cg*GSTM1-1 (pdb ID 1gsu) [37]. (**F**) GSH bound to *Lv*GSTMu (PDB id 5an1) [38]. Figure created with BIOVIA Discovery Studio.

The sequence identity of *Cd*GSTM1-1 with other mu class GSTs ranges from 50–80% (Table 4), resulting in root mean square deviations (RMSDs) between 0.44–0.85 Å and high conservation of the secondary structure elements. The structure-based sequence alignment is shown in Figure 9. Tyr116 is highly conserved in all of the compared mu class GSTs, while Met35 is found be substituted by other hydrophobic residues Ala and Val in *Gg*GSTM1-1 (PDB id 1gsu) and in LvGSTmu (PDB id 5an1), respectively. Arg43 is found to be present in *Cd*GSTM1-1, *h*GSTM1-1 (PDB id 1xw6), and *Rn*GSTM1-1 (PDB id 4gst), but is substituted by Gln, Pro, and Lys in *Mm*GSTM7, *Gg*CGSTM1-1, and *Lv*GSTM1-1, respectively. Furthermore, Met212 is found to be conserved in the *h*GSTM1, while Leu210 is replaced by Ser in the equivalent position in *h*GSTM1-1 and with Thr and Trp in *Mm*GSTM7 and *Gg*CGSTM1-1, respectively. The bound xenobiotics compound, formic acid (FMT), is found to interact via weak van der Waals forces with the GTB moiety in the G-site. The G-site for each of the compared structures is illustrated in Figure 10. Although the residues that interact with GSH are strictly conserved throughout the compared structures, some differences in the orientation of the N- and C-terminal region of bound GSH are observed (Figure 10). Nevertheless, the overall distribution of charge and the van der Walls interaction of the G-site is strictly conserved throughout the compared structures, allowing for the formation of a cavity with strictly conserved polar/hydrophobic features.

The interface comparisons of *Cd*GSTM1-1 with different mu class GSTs showed similarities (Supplementary Table S1). Approximately 16% of the total number of residues are involved in the dimer interface interactions. The solvation free energy gain upon the formation of the A−C and B−F interfaces is −8.5 and −7.8 kcal/M, respectively. A total of 22 salt bridges and 18 hydrogen bonds are formed at the interface between the A−C dimer, whereas at the B−F interface, 20 salt bridges and 17 hydrogen bonds are formed. The salt bridges between the A−C and B−F subunits are shown in Supplementary Table S2.

### 3.5. Thermal Stability of CdGSTM1-1

**Figure 11:**
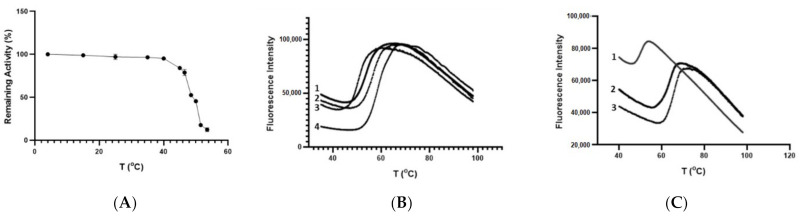
Thermal stability study for *Cd*GSTM1-1. (**A**) The kinetics of thermal inactivation curve for *Cd*GSTM1-1. The residual activities were measured after heat treatment at various temperatures (°C) for 5 min. (**B**) Thermal denaturation curves for *Cd*GSTM1-1 (2.25 μg) in the absence (line 3, T_m_: 50.4 °C) or presence of various concentrations of GSH (line 1: 0.1 mM, T_m_: 53.8 °C, line 2: 0.5 mM, T_m_: 56.1 °C, line 4: 1 mM, T_m_: 58.1 °C). (**C**) Thermal denaturation curves for *Cd*GSTM1-1 (2.5 μg) in the absence (line 1, T_m_: 50.4 °C) or presence of various concentrations of S-hexyl-GSH (line 2: 0.1 mM, T_m_: 62.9 °C, line 3: 0.5 mM, T_m_: 65.3 °C).

To evaluate the thermal stability of the enzyme, the kinetics of thermal inactivation was achieved, as shown in Figure 11A. The T_m_ value obtained was 49.09 °C ± 0.2, which agrees with the results obtained by dynamic multimode spectroscopy (DMS) [14]. Furthermore, the enzyme’s stability is comparable to that of the homologous human enzyme, suggesting the absence of any evolutional pressure from hoarse habitats on *Camelus dromedarius.*

The enzyme’s thermal stability was also investigated using differential scanning fluorometry (DSF), and the results are shown in Figure 7B,C. DSF was performed in the absence or presence of different concentrations of the natural substrate GSH (Figure 11B) and the S-substituted GSH, the S-hexyl-GSH (Figure 11C). From the results illustrated in Figure 11B,C, it is evident that the presence of GSH or S-hexyl-GSH significantly affects the enzyme’s thermal stability. A concentration dependence influence of T_m_ on the concentration of GSH or S-hexyl-GSH was observed, as illustrated in Figure 11, suggesting the formation of different types of complexes with distinct structural and thermodynamics features. Interestingly, in the presence of 0.5 mM GSH and S-hexyl-GSH, the T_m_ of the enzyme (50.4 °C) increased by 5.7 °C and 14.9 °C, respectively.

A plausible explanation for the dramatic shift of T_m_ in the presence of S-hexyl-GSH may be connected to the induced-fit mechanism operated by *Cd*GSTM1-1 (Figure 7B). The more compact and well-packaged structure upon S-p-nitrobenzyl-GSH binding, as a consequence of the ~4 Å movement of the loop β2-α2, may contribute favorably to the overall structural stability of the S-hexyl-GSH complex, compared to the free and the GSH-bound enzyme. The contribution of α-helix 2 on the thermostability of other class GSTs, such as the tau class GST4-4 from *Glycine max* has been previously observed [39].

## 4. Conclusions

In the present work, biochemical, X-ray crystallographic data and enzyme kinetics analysis were employed to aid in addressing structure−function relationships and ligand-binding features of the recombinant *Cd*GSTM1-1. Despite the high level of amino acid sequence identity, the catalytic properties of *Cd*GSTM1-1 and *h*GSTM1-1 appears to be quite different. Comparisons with the crystallographic structure of a homologous *h*GSTM1-1 indicated that several nonconserved amino acid residues play an important role in the formation of the H-site of *Cd*GSTM1-1. This suggests that diversification in the evolution of these genes has occurred primarily in the substrate binding regions to allow for the adaptation of these organisms in diverse environments, and therefore to cope with a variety of abiotic stresses caused by foreign compounds and climate conditions.

## Figures and Tables

**Table 1:** Substrate specificity of *Cd*GSTM1-1. Results represent the means of triplicate determinations, with variation less than 5 % in all cases.

Electrophile Substrates	U/mg
1-Chloro-2,4-dinitrobenzene	12.6
1-Bromo-2,4-dinitrobenzene	14.3
1-Fluoro-2,4-dinitrobenzene	8.0
1-Iodo-2,4-dinitrobenzene	1.1
p-Nitrobenzyl chloride	10.1
4-Chloro-7-nitrobenzofurazan	-
Fluorodifen	1.1
2,3-Dichloro-4-[2-methylene-butyryl]phenoxy)acetic acid (Ethacrynic acid)	-
*trans*-4-Phenyl-3-buten-2-one	-
*trans*-2-Nonenal	1.7
Cumene hydroperoxide	-
tert-Butyl hydroperoxide	-
Allyl isothiocyanate	5.4
Phenethyl isothiocyanate	5.6
2-Hydroxyethyl disulphide (2,2-dithiodiethanol)	-
Sulphanilamide	4.3
Epoxy-3-(p-nitrophenoxy)-propane	-
Bromosulfalein	-
**GSH Analogues ^a^**	
Glutathione reduced ethyl ester	6.6
γ-Glu-Cys	-
Cys-Gly	-
Cysteine	-
N-Acetyl-L-cysteine	-

**^a^** Enzyme assays were carried out under standard conditions using CDNB as the xenobiotic substrate.

**Table 2:** Steady-state kinetic analysis of *Cd*GSTM1-1 for the CDNB/GSH substrate system.

Kinetic Parameters	CDNB	GSH
K_m_ (mM)	0.84 ± 0.0994	0.0867 ± 0.0117
k_cat_ (min^−1^)	1608 ± 82	
k_cat_ /K_m_^CDNB^ (mM^−1^min^−1^)	1914.3 ± 370	

**Table 3:** X-ray crystallographic data collection and refinement statistics (numbers in parentheses refer to the highest resolution shell).

Data Collection	*Cd*GSTM1-GTB	*Cd*GSTM1-GSH
Wavelength (Å)	1.0322	1.0322
Resolution range (Å)	66.68–2.05 (2.11–2.05)	95.9–2.55 (2.64–2.55)
Space group	*P*2_1_	*P*2_1_
Unit cell	50.4 177.4 59.4 90 115.0 90	50.8 150.2 191.8 90.0 90.0 90.0
Total observations	192,763 (11609)	323,629 (30,311)
Unique reflections	55,058 (4146)	49,030 (4404)
Multiplicity	3.5 (2.8)	6.6 (6.9)
Completeness (%)	93.5 (90.5)	100 (100)
Mean I/sigma(I)	5.0 (1.4)	10.3 (0.9)
Wilson B-factor (Å^2^)	33.18	49.03
*R*_meas_	0.175 (1.593)	0.188 (2.369)
*R*_pim_	0.106 (0.969)	0.099 (1.238)
CC_1/2_	0.984 (0.305)	0.997 (0.348)
**Refinement**		
Reflections used in refinement	54,874 (5265)	48,915 (4780)
Reflections used for R-free	2627 (242)	2393 (213)
*R*_work_/*R*_free_	0.195 (0.290/0.252 (0.359)	0.206 (0.287)/0.273 (0.318)
Number of non-hydrogen atoms	7762	7564
Macromolecules	7232	7232
Ligands	127	81
Solvent	403	251
Protein residues	868	872
RMSD in bonds (Å)	0.008	0.008
RMSD in angles (°)	0.92	0.96
Ramachandran favored/allowed/outliers (%)	96.1/3.4/0.5	95.9/3.4/0.7
Rotamer outliers (%)	0.0	0.0
Clashscore	8.6	5.8
Average B-factor (Å^2^)	55.6	59.1
Macromolecules	56.2	59.1
Ligands	47.2	74.0
Solvent	47.3	54.2
Number of TLS groups	4	30
PDB id	7opy	7opz

**Table 4:** Comparison statistics of *Cd*GSTM1 with other µ-class GSTs.

µ-GSTs	Organisms	PDB Entries	RSMD Values
*h*GSTM1	*Homo sapiens*	1xw6	0.44
*h*GSTM2	*Homo sapiens*	2ab6	0.53
*h*GSTM4	*Homo sapiens*	4gtu	0.73
*Mu*GSTM7	*Mus musculus*	2dc5	0.79
*Gg*GSTM1-1	*Gallus gallus*	1gsu	0.72
*Lv*GSTMu	*Litopenaeus vannamei*	5an1	0.85
*Rr*GSTMu	*Rattus rattus*	6gst	0.50

## Data Availability

Data are contained within the article or supplementary material.

## Supplementary Materials

The following are available online at https://www.mdpi.com/article/10.3390/life12010106/s1, Table S1. Comparison of the subunit−subunit interface area; Table S2. Salt bridges between the *Cd*GSMT1 subunits (cut-off distance 4.0 Å).
