# Supplementary material for: Structural and Functional Characterization of Camelus dromedarius Glutathione Transferase M1-1
Source: Life (Basel). 2022 Jan 12;12(1):106. doi: 10.3390/life12010106 (PMC8780062; doi:10.3390/life12010106)
Supplement: Supplementary file 1 [file life-12-00106-s001.zip › life-1504299-supplementary.pdf]

life

MDPI

Supplementary

Table S1. Comparison of the subunit–subunit interface area.

μ-GST	Interface Area (Å²)
*Cd*GSTM1-1	1379.5
1xw6	1249.2
1ab6	1383.6
4gtu	1353.6
2dc5	1358.8
1gsu	1212.2
5an1	1157.5
6gst	1328.5

Table S2. Salt bridges between the *Cd*GSMT1 subunits (cut-off distance 4.0 Å).

Subunit C	Distance (Å)	Subunit A
Arg82 NE	3.47	Glu91 OE1
Arg82 NH2	3.21	Glu91 OE1
Arg82 NE	3.00	Glu91 OE2
Arg82 NH2	3.99	Glu91 OE2
His68 NE2	3.18	Glu92 OE1
Arg78 NH2	2.98	Asp98 OD1
Arg78 NE	3.10	Asp98 OD1
Arg78 NH2	3.59	Asp98 OD2
Arg78 NE	2.74	Asp98 OD2
Arg82 NH1	2.86	Asp98 OD2
Arg82 NH2	3.81	Asp98 OD2
Glu91 OE1	3.97	Arg82 NH2
Glu91 OE1	3.17	Arg82 NE
Glu91 OE2	2.76	Arg82 NH2
Glu91 OE2	3.02	Arg82 NE
Asp98 OD1	3.00	Arg78 NH1
Asp98 OD2	2.83	Arg82 NH1
Asp98 OD2	3.99	Arg82 NH2
Glu101 OE1	3.27	Arg78 NH1
Glu101 OE1	3.94	Arg78 NH2
Glu101 OE2	3.96	Arg78 NH1
Glu 101 OE2	3.24	Arg78 NH2
**Subunit F**	**Distance (Å)**	**Subunit B**
Arg78 NE	3.20	Asp98 OD1
Arg78 NE	2.95	Asp98 OD2
Arg78 NH2	3.27	Asp98 OD1
Arg78 NH2	3.89	Asp98 OD2
Arg82 NE	2.85	Glu91 OE1
Arg82 NE	3.09	Glu91 OE2
Arg82 NH1	3.06	Asp98 OD2
Arg82 NH2	3.70	Glu91 OE1

Arg82 NH2	2.60	Glu91 OE2
Glu91 OE1	2.98	Arg82 NE
Glu91 OE2	3.16	Arg82 NE
Glu91 OE2	2.90	Arg82 NH2
Glu92 OE2	3.68	His68 NE2
Asp98 OD1	2.77	Arg78 NH1
Asp98 OD2	2.83	Arg82 NH1
Asp98 OD2	3.80	Arg82 NH2]
Glu101 OE1	3.27	Arg78 NH1
Glu101 OE1	3.85	Arg78 NH2
Glu101 OE2	3.89	Arg78 NH1
Glu101 OE2	3.08	Arg78 NH2
